# Dr. Frederic William Bartlett

**Published:** 1897-04

**Authors:** 


Dr. Frederic William Bartlett, of Buffalo, died at his residence in this city, No. 523 Delaware avenue, March 17, 1897, aged 71 years. Dr. Bartlett was born at Kingston, Mass., and his parents, Uriah and Olive (Holmes) Bartlett, were of old pilgrim stock, both having been lineal descendants of Elder William Brewster, Captain Myles Standish and John Alden, of the Mayflower pilgrims. Dr. Bartlett received his preliminary education at Bridgewater, taught school for a number of years, and then went South to take charge of the Lafayette Academy in Georgia. In 1846 he published the *Atlanta Luminary*, which afterward became the *Georgia Tribune.*

The agitation of the slavery question in 1848 led him to return to his native state, where he became one of the publishers of the *Old Colony Reporter* at Brocton, Mass. He began the study of medicine at New York in 1850, received his doctorate degree from the New York College of Medicine in 1854, and took up his residence at Buffalo in 1855, where he continued to live until his death. He married Adelia, daughter of Dr. James Hunter, of Whitby, Ont., December 28, 1854, who, together with his only son, Dr. G. Hunter Bartlett, survives him.

Dr. Bartlett was elected to membership in the medical society of the county of Erie in 1865 and was president in 1895 ; president of the Buffalo medical and surgical association in 1884 ; president of the Buffalo physicians' protective association in 1893–1894 ; trustee of the Buffalo academy of medicine 1893–1896 ; consulting physician to the Buffalo hospital sisters of charity 1895, and besides held several offices in nonprofessional societies. The funeral services were conducted by the Rev. J. A. Register, of St. Paul's cathedral, on Friday, March 19th, at which the medical society of the county of Erie attended in a body.

The honorary bearers were Drs. John Cronyn, Herman Mynter, R. E. Campbell, E. C. W. O'Brien, F. S. Crego, J. B. Coakley and Lucien Howe. The active bearers were Drs. John Parmenter, J. N. Goltra, A. L. Benedict, William C. Krauss, John H. Pryor, Allen A. Jones, William H. Thornton and C. R. Jewett. The burial was at Forest Lawn.

In another column the many excellent qualities of this esteemed physician are fittingly eulogised by his colleagues, who were intimately associated with him for many years.

